# Use of accounting concepts to study research: return on investment in XSEDE, a US cyberinfrastructure service

**DOI:** 10.1007/s11192-022-04539-8

**Published:** 2023-02-14

**Authors:** Craig A. Stewart, Claudia M. Costa, Julie A. Wernert, Winona Snapp-Childs, Marques Bland, Philip Blood, Terry Campbell, Peter Couvares, Jeremy Fischer, David Y. Hancock, David L. Hart, Harmony Jankowski, Richard Knepper, Donald F. McMullen, Susan Mehringer, Marlon Pierce, Gary Rogers, Robert S. Sinkovits, John Towns

**Affiliations:** 1 National Center for Supercomputing Applications, University of Illinois at Urbana-Champaign, Urbana, IL USA; 2 Department of Computer Science, Indiana University, Bloomington, IN USA; 3 Pervasive Technology Institute, Indiana University, Bloomington, IN USA; 4 Texas Advanced Computing Center, University of Texas at Austin, Austin, TX USA; 5 Pittsburgh Supercomputing Center, Carnegie Mellon University, Pittsburgh, PA USA; 6 Kelley School of Business, Indiana University, Bloomington, IN USA; 7 LIGO Laboratory, California Institute of Technology, Pasadena, CA USA; 8 Computational and Information Systems Lab, University Corporation for Atmospheric Research, Boulder, CO USA; 9 Center for Advanced Computing, Cornell University, Ithaca, NY USA; 10 National Institute for Computational Sciences, University of Tennessee at Knoxville, Knoxville, TN USA; 11 San Diego Supercomputer Center, University of California San Diego, La Jolla, CA USA

**Keywords:** XSEDE, TeraGrid, Cyberinfrastructure, Supercomputing, HPC, eScience, Return on Investment, Cost efficiency, Cloud computing in research, COVID-19, 68U99, 91B44, 91B18, I23, M15, O31, O32, O38

## Abstract

This paper uses accounting concepts—particularly the concept of Return on Investment (ROI)—to reveal the quantitative value of scientific research pertaining to a major US cyberinfrastructure project (XSEDE—the eXtreme Science and Engineering Discovery Environment). XSEDE provides operational and support services for advanced information technology systems, cloud systems, and supercomputers supporting non-classified US research, with an average budget for XSEDE of US$20M+ per year over the period studied (2014–2021). To assess the financial effectiveness of these services, we calculated a proxy for ROI, and converted quantitative measures of XSEDE service delivery into financial values using costs for service from the US marketplace. We calculated two estimates of ROI: a Conservative Estimate, functioning as a lower bound and using publicly available data for a lower valuation of XSEDE services; and a Best Available Estimate, functioning as a more accurate estimate, but using some unpublished valuation data. Using the largest dataset assembled for analysis of ROI for a cyberinfrastructure project, we found a Conservative Estimate of ROI of 1.87, and a Best Available Estimate of ROI of 3.24. Through accounting methods, we show that XSEDE services offer excellent value to the US government, that the services offered uniquely by XSEDE (that is, not otherwise available for purchase) were the most valuable to the facilitation of US research activities, and that accounting-based concepts hold great value for understanding the mechanisms of scientific research generally.

## Introduction

Since at least as far back as 1912, economists have worked to understand the relationships among economic investment in innovation, the results of investment, and the economic benefits of investment (Schumpeter, 2003). An understanding of the relationship between investment in research and subsequent outcomes is critical to understanding the mechanisms of science. Today, much scientific research depends upon information technology (IT) resources. Understanding the mechanisms of science thus particularly requires an interrogation of the effects of investments in IT that support science. Existing reports convincingly, but only generally, relate government investment in broad areas of IT research to broad areas of economic benefit and improved quality of life (e.g. National Academies of Sciences, 2020). In order to understand the details of the impacts of IT investments on science, it is necessary to analyze the details of particular IT facilities and services.

XSEDE—the eXtreme Science and Engineering Discovery Environment—is an IT service designed to support research across all disciplines of science and engineering in the US (Towns et al., 2014). It is an example of what the US National Science Foundation (NSF) terms cyberinfrastructure. Similar but not identical to the European term eScience, cyberinfrastructure is defined as “computing systems, data storage systems, advanced instruments, data repositories, visualization environments, and people, all linked together by software and high performance networks to improve research productivity and enable breakthroughs not otherwise possible” (Stewart et al., 2019). Cyberinfrastructure includes computational resources such as supercomputers and clouds. XSEDE is among the largest and longest-running cyberinfrastructure projects ever funded by the NSF. Through two rounds of funding, the NSF has invested US$257,465,523 (National Science Foundation, 2011, 2016) in XSEDE. XSEDE started on 1 July 2011 and ceased operations on 31 August 2022, making the average expenditure US$23.4M per year. (Financial figures hereafter given in US$.)

XSEDE’s primary goal is to aid the advancement of science and engineering research; it assists and enables researchers to perform sophisticated data analyses and computer simulations and to create visualizations. XSEDE also provides training and consulting to aid people in using advanced cyberinfrastructure systems. The researchers that XSEDE supports range from new graduate students with no formal training in computational science to some of the most sophisticated computational scientists in the world.

Its size and duration made XSEDE an ideal project to study in order to understand how investments in cyberinfrastructure affect the mechanisms of science. Analyzing the value of public investments in national advanced computing infrastructure is particularly useful at this point in time, when governments and researchers have multiple potential sources of advanced computing facilities, including commercial cloud computing services.

When analyzing government investments in cyberinfrastructure and advanced IT services as a way to support research and foster innovation, three questions are worthy of consideration: (1) in the long term, has investment in a particular project yielded proportionally beneficial results to the taxpayer public who funded that research?, (2) in the short term, are the information technology services supported by governmental funds delivered in financially efficient ways?, and (3) how does government investment in cyberinfrastructure affect the mechanisms of scientific research? We focus here on the second and third of these questions. In particular, we carry out a quantitative analysis of the value of the US government’s investment in XSEDE to determine whether XSEDE services are delivered in a financially effective way. Based on this analysis, with some additional data about XSEDE’s operations, we consider how investment in XSEDE has affected the mechanisms of scientific research in the US. In doing so, we intend to demonstrate that concepts from the field of accounting can be used as tools in understanding the mechanisms of science. We hope this will encourage others to take this type of approach to questions in the study of science.

## Background

### What is XSEDE?

The NSF began providing advanced IT services to the US national research community in the 1980s with what it called the Supercomputer Centers Program (Stewart et al., 2019). This program funded four centers that provided supercomputer resources to the US research community. Access to these systems was granted competitively, based on an application process, to allocate the limited resources available, but without financial cost to the researchers using the systems. At that time there was little consideration of the cost-benefit analysis. The NSF justified investments in these centers on the basis of scientific achievements that could be achieved by no means other than through the use of supercomputers. The NSF’s approach to delivering what are now called cyberinfrastructure services to the US has evolved considerably since the 1980s.

For the last decade, the NSF has funded two related but distinct types of cyberinfrastructure services: XSEDE, serving as a general “front door” and support mechanism; and service providers (SPs), which operate cyberinfrastructure resources such as supercomputers, high performance computing (HPC) clusters, storage systems, and visualization systems. The resources operated by these SPs are available to the US research community for purposes of non-classified and non-commercial research. Grant awards for operating SP facilities typically last for six years. The NSF regularly makes new awards for such resources, and older resources are routinely retired, so the cyberinfrastructure resources available to the US research community are consistently updated and altered.

The most valuable of the services offered by XSEDE and by SPs are allocated through a process in which principal investigators (PIs) apply for resources such as computer time or expert consultant time. These resources, like the allocations of computer time on the original NSF supercomputer centers, are given out without cost to PIs, although demand typically exceeds supply of resources. In the case of computer time, demand as measured by proposals is several times the amount available.

**Fig. 1:**
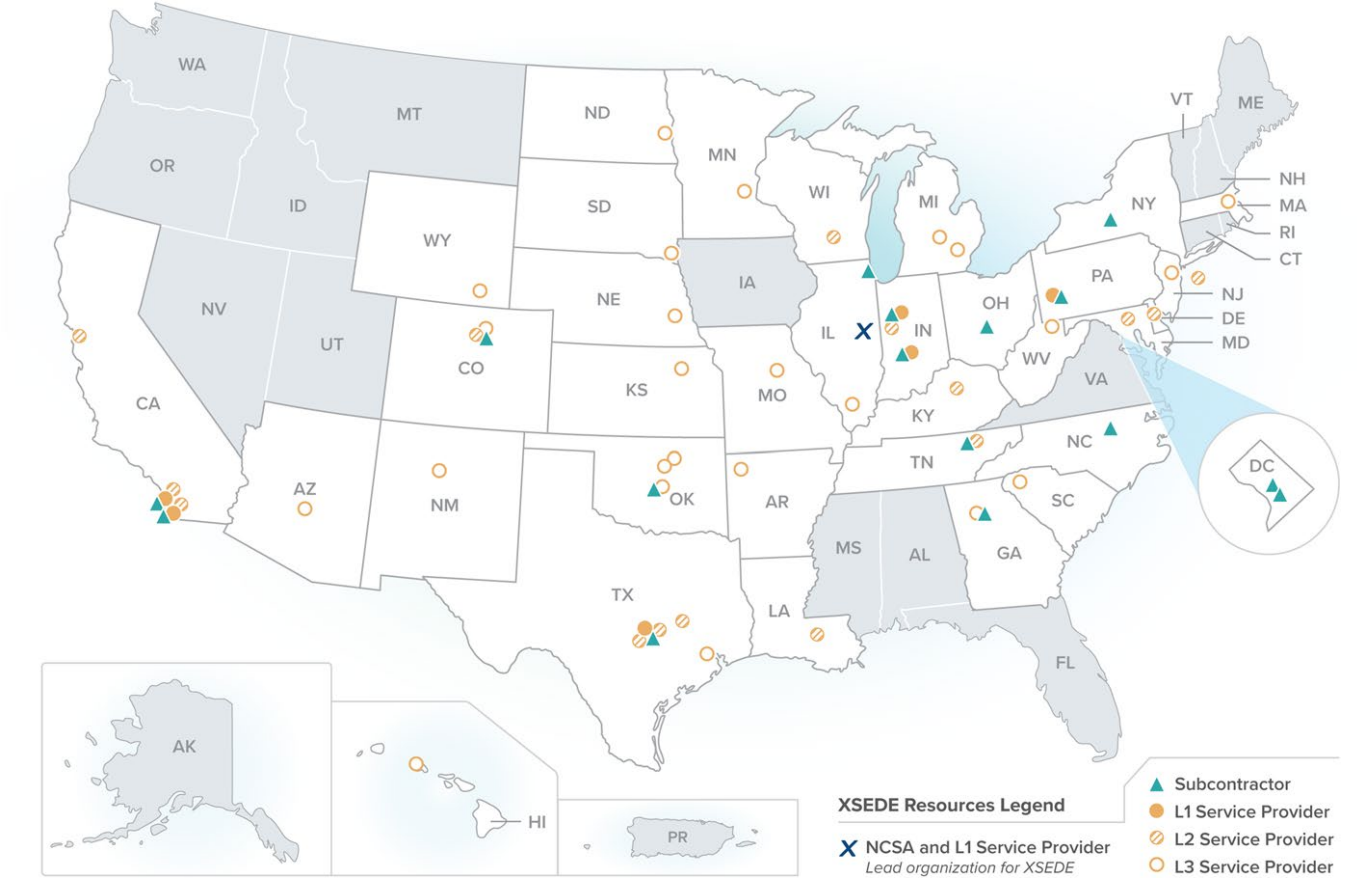
Distribution of XSEDE SPs and subcontractors throughout the US (States and territories shaded in gray didn’t have SPs or subcontractors)

The following statistics provide a sense of the size and scope of XSEDE:

- XSEDE supported a total of 15 supercomputers and HPC clusters and one cloud system with an aggregate processing capability of 28.6 petaFLOPS (XSEDE, 2022a). (One petaFLOPS is one quadrillion floating-point mathematical operations per second.)
- XSEDE employed a total of 212 individuals for a total full-time equivalent (FTE) of 91.8 individuals.
- XSEDE directly supported more than 11,000 researchers and students, including individuals in every state in the US, the District of Columbia, Puerto Rico, Guam, and the US Virgin Islands. (These are people who logged directly into an XSEDE-supported system in PY10.) More than 18,000 other individual users are supported indirectly via Science Gateways, web-based interfaces customized for particular analysis and simulation tasks (Stewart et al., 2019).
- Thirty-three states and the District of Columbia have been home to an SP, an XSEDE subcontractor, or both, as shown in Fig. 1.

There is one additional layer of complexity in this service ecosystem. SPs are classified into one of three levels (XSEDE, 2022b):

- *Level 1 SPs (L1 SPs)*. L1 SPs receive federal funding to operate large systems such as the Stampede, Bridges, and Comet supercomputers and the Jetstream cloud system. L1 SPs are required by the terms of their NSF funding to integrate their systems with and manage allocation and accounting processes for the national community through XSEDE.
- *Level 2 SPs (L2 SPs)*. L2 SPs have formal allocation processes and are available to a broad audience, but are not required to adhere to XSEDE operational practices and are not necessarily allocated through XSEDE.
- *Level 3 SPs (L3 SPs)*. L3 SPs do not allocate use of resources outside their own institutions but wish to make them known to the national community.

**Fig. 2:**
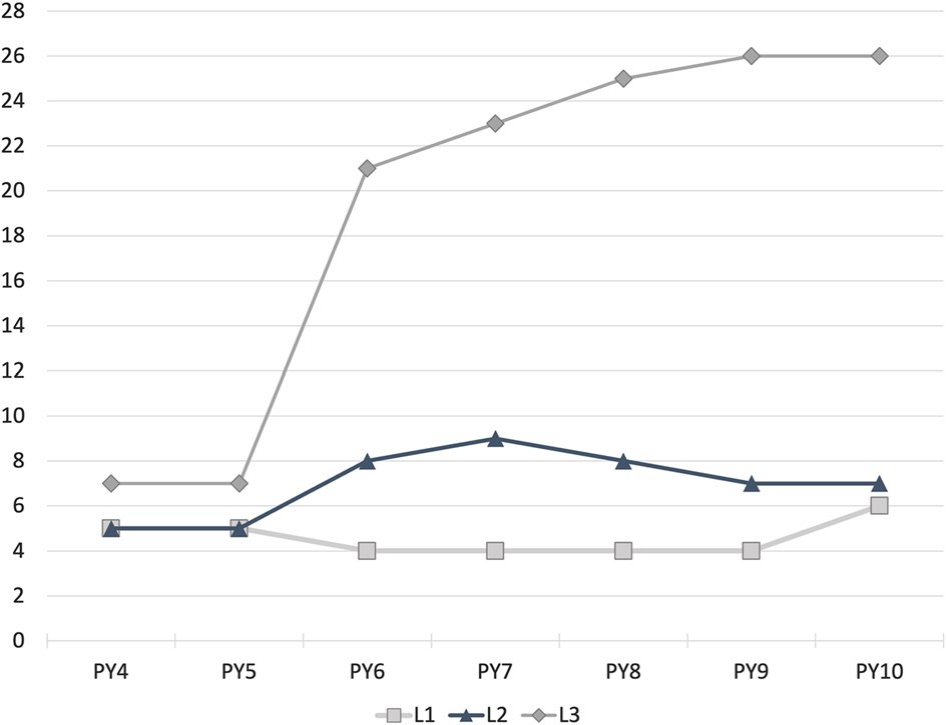
Number of level 1, 2, and 3 service providers from Program Year 4 (2014) through Program Year 10 (2021)

L1 SPs are established by the NSF through grant proposal solicitations, so the number of L1 SPs is determined by the NSF. Organizations apply to XSEDE to acquire status as an L2 or L3 SP, explaining their interest in an affiliation. There is no predetermined number of L2 and L3 SPs. Figure 2 shows the number of SPs at each level over the time period considered in this report.

### Scientometric and financial analyses of IT services

There have been several bibliometric analyses of XSEDE. Von Laszewski and his coauthors performed a bibliometric analysis and demonstrated that papers that utilize XSEDE resources are cited statistically significantly more often than comparable publications that do not (von Laszewski et al., 2015). Knepper and Börner (Knepper & Börner, 2016) analyzed the usage of XSEDE-supported resources and found that researchers who used the most computing time tended to be from disciplines with a long tradition of use of supercomputers, such as astronomy and physics. Kee studied the TeraGrid, the predecessor to XSEDE, and clarified the dynamics of operating a large, grant-funded IT facility (Kee & Browning, 2010). The first peer-reviewed paper we have found addressing the financial effectiveness of investments in IT dates to 1962 (Saunders & Jones, 1992). Apon et al. (2015) and Smith and Lien (2021) demonstrated that increased investment in cyberinfrastructure leads to an increase in desired outcomes, including peer-reviewed publications, grant revenue, doctoral degrees conferred, and university rankings. Stewart et al. (2015) developed the ROI analysis methods used in this paper. Stewart, Wernert, et al. (2022) reviewed the existing literature regarding ROI analyses of cyberinfrastructure.

### Assessing ROI for cyberinfrastructure

The following explanation is based on Stewart et al. (2019). The textbook definition of ROI is “a ratio that relates income generated... to the resources (or asset base) used to produce that income” (Kinney & Raiborn, 2011), or:

$$\begin{aligned} \text{ROI} = \dfrac{\text {Funds received from sales or services}}{\text {Costs to deliver sales or services}} \end{aligned}$$

However, the services offered by XSEDE are made available without cost to the national research community, so there is no revenue per se. To create a measure conceptually similar to ROI, where value created is compared to the monetary investment, Stewart et al. (2019) coined the term ROI_proxy_:

$$\begin{aligned} \text {ROI}_\mathrm{proxy}=\dfrac{\text {Market value of services delivered and products created}}{\text {Cost to deliver services and products}} \end{aligned}$$

**Fig. 3:**
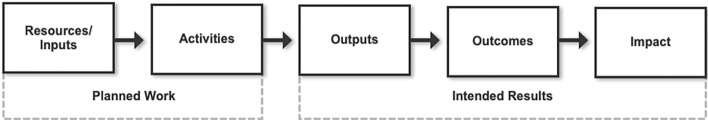
Logic model of organizational processes

Figure 3 shows a logic model of organizational processes based on a report by the WK Kellogg Foundation (2004). ROI_proxy_ can be applied to any or all of the “Activities,” “Outputs,” and “Outcomes” blocks. We focus on the “Activities” block of the organizational process model by comparing the market value of services delivered by an organization with the actual cost of the services delivered. This enables analysis of the financial effectiveness of a cyberinfrastructure service such as XSEDE.

## Materials and methods

**Fig. 4:**
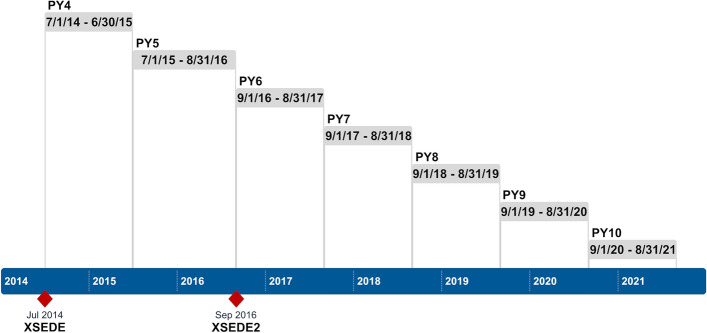
Start and end dates of the XSEDE project years

We collected data to assess ROI_proxy_ for government investment in XSEDE from 2014 through 2021. The XSEDE project includes two separate NSF funding actions (XSEDE in 2011, XSEDE2 in 2016) (National Science Foundation, 2011, 2016). Project Year (PY) 5 was 14 months long, at the specification of the NSF. The start and end dates of each year are shown in Fig. 4. Our data collection and analysis methods are summarized below and described extensively in Wernert et al. (2022). The actual data are also available in Costa et al. (2022), enabling anyone who wishes to reanalyze these data.

### XSEDE services to which we can attach a value

XSEDE services to which we can attach a market value are described below:

- *Operational and administrative support for SPs*:
  - *Services for level 1 SPs.* These include: review and management of applications for allocation of available SP resources; account management and tracking of resource use against allocations; software for integration of SPs into the XSEDE ecosystem; educational materials related to SP resources; and dissemination of information about SP resources and services.
  - *Services for level 2 and level 3 SPs.* XSEDE provides substantially fewer services specifically for L2 and L3 SPs. L2 and L3 SPs have the option of using XSEDE-created software and often do so. In some cases, XSEDE operates allocation processes for L2 SPs. As noted, L2 and L3 SPs are not required to integrate with XSEDE systems and policies.
  - *Cybersecurity services.* XSEDE provides cybersecurity services protecting SPs and XSEDE resources as a whole.
- *Services related to scientific software and cyberinfrastructure system administration.* These services are of use to SPs, colleges and universities that are not service providers, and individual researchers and students:
  - *Data transfer.* XSEDE supports fast, secure, reliable data transfer for SPs and for individuals using XSEDE-supported services by contracting for data transfer services from Globus Online (Globus, 2022).
  - *Software repository.* XSEDE provides a software repository used by system administrators, individual researchers, and students to download and install software on local campus cyberinfrastructure systems (Navarro et al., 2017).
  - *Software optimization.* XSEDE regularly improves the speed and function of open source software and contributes optimized code back to the relevant software repositories (Wilkins-Diehr et al., 2015).
  - *Campus visits.* XSEDE provides on-site and/or remote campus consultations to install (or reinstall) software on local campus cyberinfrastructure resources in order to create a system largely consistent with XSEDE-allocated systems (Coulter et al., 2016).
- *XSEDE support services.* These are of use to individual researchers and students, colleges and universities, and SPs:
  - *Training services.* Training is offered in person, via live webinars, and in online asynchronous tutorials. Some classes include assessment of student learning, with successful completion resulting in a badge certificate.
  - *Helpdesk services* (trouble ticket resolution). This involves resolving routine system use problems, such as password resets.
  - *Extended consulting services.* This is an allocated service requested by PIs, just like computer time, through an XSEDE-operated review process. Extended consulting services involve expert staff assigned to work with research group leaders and their teams to address challenging software problems. Such in-depth support often involves modifying software to run effectively on XSEDE systems (which often represent the newest system and processor architectures available). Typically, multiple months of person effort are allocated per project (Wilkins-Diehr et al., 2015).

### Assessing the financial value of XSEDE services

We assessed a “market value of services delivered” by XSEDE–focusing on the cost effectiveness of the “Activities” aspect of XSEDE organizational processes as depicted in Fig. 3 above. Market value of services delivered was calculated by measuring units of a service delivered and multiplying by a unit value derived from a published or otherwise reasonable source. Table 1 lists services for which we have measurements.

We took two different approaches to assigning values to services delivered—which we refer to as the Conservative Estimate and the Best Available Estimate. The Conservative Estimate is the approach we took in earlier works (e.g., Stewart et al. 2015, 2019). All of the valuations used in the Conservative Estimate are based on valuations that are published openly and represent means or lower bounds of available relevant data. For example, as a value for training activities about how to use supercomputers, we used the hourly cost of training for MS Excel®. The Conservative Estimate of ROI_proxy_ for XSEDE thus represents a lower bound on the ROI for government investment in XSEDE and has proved particularly useful in discussions of science policy and funding. Table 2 shows the per unit valuation assigned for XSEDE services. Then, in order to have ROI_proxy_ estimates that are less biased toward the conservative side, we have also created what we call a Best Available Estimate of ROI_proxy_. We use this phrase because the valuations used are indeed the best estimates that we feel can be justified based on market prices in the US. For example, for online training we use the costs for Red Hat Enterprise Linux training, which is roughly on the same level of sophistication as the training XSEDE offers. The Best Available Estimate analysis uses some valuations based on services procured via contracts with terms known to the authors but not openly published.

We are not in general considering counterfactual questions such as “what would have happened had the US government not invested in XSEDE at all?” Rather, we focus on the question “Given that the US government has actually invested in XSEDE, has this investment proved to be a good one on behalf of the US government and its taxpayers?” and on what we can learn about science from financial analyses of XSEDE.

**Table 1:** Summary of XSEDE services for which there are financial valuations

Service	Recipients	Measures for which there are data and financial valuations
*Operational and administrative support for SPs*		
Operational & administrative support for SPs	L1/L2/L3 SPs	Measure: How much effort the SP would have had to invest in FTEs from its own staff in the absence of XSEDE services
		Assessed by: Survey
		Converted to $ by: Multiply FTE-year equivalents times average annual salary & benefits for XSEDE staff
Cybersecurity services	XSEDE & L1 SPs	Measure: used only in best available estimates
		Assessed by: Cost of one cyberattack avoided
*Converted to $ by: Published costs of cybersecurity breaches*		
*Services related to scientific software and cyberinfrastructure system administration*		
Data transfer	SPs, colleges, universities, individuals	Measure: GB of data moved
		Assessed by: File movement software tabulates volume moved
		Converted to $ by: Use fees based on AWS (Amazon Web Services)
Software repository	SPs, colleges, universities, individuals	Measure: number of downloads to US IP addresses
		Assessed by: Counts of software module downloads measured by software distribution server
		Converted to $ by: (1) For Conservative Estimate, we estimate each download saves 0.5 h × national average system administrator salary & benefits; (2) For Best Available Estimate, we estimate each download saves 1 hour at an hourly cost of $250
Software optimization	SPs, colleges, universities, individuals	Measure: Value of system hardware expenses avoided as a result of XSEDE services
		Assessed by: Acquisitions costs reports from XSEDE and LIGO
		Converted to $ by: (1) For Conservative Estimate, we use costs avoided by not buying extra hardware for LIGO; (2) For Best Available Estimate, add 10% of the hardware costs for the L1 SPs
Campus visits	Colleges, universities	Measure: number of FTE-months PI’s group would have had to put in themselves in the absence of XSEDE campus visit
		Assessed by: Interviews with PIs
		Converted to $ by: (1) For Conservative Estimate, multiply time saved by national average system administrator salary & benefits (2) For Best Available Estimate, time saved multiplied by $250/h
*XSEDE support services*		
Training services	Educators, researchers, students, other individuals	Measure 1: Hours of training (on demand, remote, live)
		Assessed by: Reports of training event attendance
		Converted to $ by: (1) For Conservative Estimate, costs for Microsoft Excel training; (2) For Best Available Estimate, costs for training for RedHat Enterprise Linux
		Measure 2: Number of badges awarded
		Assessed by: Reports from training management system
		Converted to $ by: $1,000/badge, based on market rates of $2600 adjusted down because XSEDE badges are less known than others
Helpdesk services	Primarily individuals	Measure: number of tickets
		Assessed by: Reports from trouble ticket management system
		Converted to $ by: Multiply by national average cost of trouble ticket
Extended consulting	Principal investigators	Measure: Number of person-months the PI’s group would have had to put in themselves without XSEDE (truncated at maximum of 24)
		Assessed by: PI interviews to determine FTE-months saved
		Converted to $ by: (1) For Conservative Estimate, multiply time saved by national average computational scientist salary & benefits (2) For Best Available Estimate, time saved multiplied by $250/h

#### Measurement of service delivery

##### Directly measured services

Several services are measured as they are delivered. The amount of data moved, number of software modules downloaded, number of tickets resolved, and number of hours of online training delivered are all measured by the automated systems that deliver these services. Attendance at in-person training and number of badges awarded are derived from electronic records of course registration and testing results.

##### Surveys of service recipients

Surveys of service recipients were used to evaluate XSEDE services that have no equivalent in the marketplace. This includes services delivered to SPs, extended consulting services, and campus visits.

*Surveys of SPs.* To measure value to SPs, an authorized representative of each SP was asked via a survey the following question about each of several services provided by XSEDE: “Considering each of the following XSEDE-provided services, what value did your SP receive during [this] project year? Please express your response in terms of the FTE effort that would have been required for your SP to provide the service.” Estimates of FTE value equivalents were requested to a degree of precision no more fine-grained than 0.25 FTE years. The contents of the online survey are included in Wernert et al. (2022). Survey data were collected by an independent survey organization within Indiana University, with advance approval by Indiana University’s Institutional Review Board.

*Interviews with recipients of extended consulting services.* Extended consulting services offered to PIs and lab groups (Wilkins-Diehr et al., 2015) were estimated in a way similar to the analysis of value to SPs. After a project was completed, XSEDE staff asked research group leaders (or appropriate representatives), “Had you not had XSEDE consulting support, can you estimate how many person-months it would have taken to get to the same stage that your project achieved with the XSEDE support?” With some regularity, we received answers of “I could not have done this at all without XSEDE’s help,” or a number greater than 24 months. To keep our calculations conservative, we estimated the value of XSEDE assistance with a maximum value of 24 months—including when a researcher indicated that they could not estimate a time savings because they simply could not have completed the project without the time and expertise provided by XSEDE consultants.

*Interviews with representatives of institutions receiving campus visits.* The value of campus site visits in which XSEDE staff members assist in installing or reinstalling campus-based cyberinfrastructure resources was estimated by interviewing representatives of institutions that had received such visits.

#### Financial values of services

In general, the dollar value of services was estimated by multiplying the number of units of the services by a per unit value. These unit values are detailed in Costa et al. (2022) and summarized in Table 2. A few items merit extended explanations.

*Staff costs.* A general difference between the Conservative Estimate and Best Available Estimate is the value assigned to staff time. In the Conservative Estimate, salaries for systems administrators and software consultants are taken from average values found in national surveys. This is conservative because the systems administrators and consultants performing these roles in XSEDE are very much the “best of the best,” not average. Thus for the Best Available Estimate, we use a value of $250 per hour (and 2000 work hours per year) as the value of systems administrator and consultant time, based on recent contracts signed by Indiana University with terms known to the authors but not published openly.

*Cybersecurity services.* There are no standard values for cybersecurity services published anywhere that we can find. For the Conservative Estimate of ROI_proxy_ we simply did not include a value for this service provided by XSEDE. For the Best Available Estimate for ROI_proxy_ we took what we believe to be a more realistic approach based on experiences from TeraGrid, the predecessor of XSEDE. During the time between 2004 and 2011, TeraGrid managed services including cybersecurity for what are now called level 1 SP resources, and there was one system break-in that resulted in the disruption of system operations. (This hack affected systems of significance in terms of national security interests and was not publicly documented. Several of the authors of this paper were directly involved in the response to this event.) There were no such break-ins at any time during the operation of XSEDE. IBM estimated the cost of a system break-in at $3.86M (IBM, 2021). The cost of a break-in at an academic research site handling only non-sensitive data is certainly below this. For the Best Available Estimate analysis of ROI_proxy_, we used an estimate of $1M as the value of one break-in, avoided because of the high quality of XSEDE services, that might otherwise have taken place.

*Data transfer services.* The data transfer services offered by XSEDE are sophisticated and unusual in their speed, capacity, security, and reliability. No commercial entities provide analogous services. The closest commercial service for which there exist published rates is AWS. These rates are the same as or lower than those of other commercial cloud providers.

*Software repository.* We estimated the value of the XSEDE software repository by estimating that each automated installation of a piece of software using the XSEDE Community Software repository saved a systems administrator at least 30 minutes of effort for the Conservative Estimate, and 60 minutes for the Best Available Estimate. (Coauthors who are or have been systems administrators, and several systems administrators we talked with within XSEDE and the research community, find these values reasonable.)

*Software optimization services.* The value of the optimization of software performance takes many forms. Most have to do with accelerating the research and discovery progress. Sometimes accelerating software can be an alternative to spending more on systems hardware. This can be the case when the available systems hardware is constrained (running at full capacity) and the only ways to increase the ability of a system to complete analyses are to purchase more hardware or make the software more efficient. To calculate an equivalent value of “systems hardware costs avoided” in the latter case, one must know how much computing resource was used to run a program and how much the optimization sped up execution of the program. In general, we do not have all of the data required to perform this calculation. For the time period considered, there is one case where we can confidently estimate the value of system costs avoided due to software enhancements. The Laser Interferometer Gravitational-Wave Observatory (LIGO) (LIGO Caltech, 2022) was designed specifically for direct detection of gravitational waves predicted by Einstein’s General Theory of Relativity. LIGO project leaders realized in 2013 that they lacked sufficient hardware resources to analyze all collected data, and estimated that purchasing the necessary equipment would cost around $75M. The NSF was unwilling to fund acquisition of that much new hardware and instead directed LIGO to seek assistance from XSEDE. A collaboration between XSEDE and LIGO resulted in changes to LIGO workflows and software optimization strategies, allowing LIGO to complete the data analysis without additional investment in hardware. Leaders of this effort from LIGO and XSEDE are among the coauthors of this paper (Couvares and Towns, respectively). They agree that LIGO and XSEDE staff deserve equal credit for the software improvements. Estimating the value of XSEDE’s contributions to LIGO as one half of the cost of the hardware that would otherwise have been needed, XSEDE’s contributions would be valued at $37,500,000. This work was done during project year 4 (PY4) of XSEDE and this value is included in the Conservative Estimate for ROI_proxy_. For the Best Available Estimate we added an additional value relative to the L1 SPs. XSEDE L1 SPs are chronically oversubscribed. The L1 SPs thus constitute an environment in which speeding up software is equivalent to having more hardware. We estimated that without XSEDE, it would have taken an average of at least 10% more hardware to run the work that was done using XSEDE L1 SP resources each year. This seems reasonable based on the experience of the authors who are directly involved in XSEDE.

*Training services.* The value of training is based on published costs for training in Microsoft Excel for the Conservative Estimate and on the basis of Red Hat Enterprise Linux systems administration training for the Best Available Estimate analysis. The latter is a training generally offered in the US that is similar in complexity to the training offered by XSEDE.

**Table 2:** Service valuations and costs of XSEDE services (values from Costa et al., 2022)

Basis for costs and services valuation	Cost measure	PY4	PY5	PY6	PY7	PY8	PY9	PY10
		Jul14–Jun15	Jul15–Aug16	Sep16–Aug17	Sep17–Aug18	Sep18–Aug19	Sep19–Aug20	Sep20–Aug21
*Operational and administrative support for SPs*								
Operational and administrative support for L1/L2/L3 SPs	Staff cost basis: average annual salary of XSEDE staff	Conservative Estimate and Best Available Estimate						
		$207,785	$211,120	$200,434	$214,468	$219,542	$210,059	$221,349
Cybersecurity services	Cost of an avoided break-in spread across the period ($1M divided by 7)	Conservative Estimate						
		$0	$0	$0	$0	$0	$0	$0
		Best Available Estimate						
		$142,857	$142,857	$142,857	$142,857	$142,857	$142,857	$142,858
*Services related to scientific software and cyberinfrastructure system administration*								
Data transfer	AWS cost	Conservative estimate and Best Available Estimate						
		$0.05/GB—using lowest and most recent value for all years						
Software repository	0.5 h at: National average annual cost for a system administrator	Conservative Estimate						
		$82,200	$84,500	$86,340	$87,070	$88,410	$89,460	$89,460
		+ 47%	+ 50%	+ 50%	+ 50%	+ 51%	+ 51%	+ 51%
		Benefits	Benefits	Benefits	Benefits	Benefits	Benefits	Benefits
	1 hour at: Hourly consultant fee	Best Available Estimate						
		$250	$250	$250	$250	$250	$250	$250
Software optimization	Cost of system hardware purchases avoided	Conservative Estimate						
		$37,500,000	$0	$0	$0	$0	$0	$0
		Best Available Estimate						
		$39,247,276	$1,678,585	$1,565,470	$1,350,980	$1,350,980	$1,350,980	$1,214,964
Campus visits	Length of visit (h): National average annual cost for a system administrator	Conservative Estimate						
		$82,200	$84,500	$86,340	$87,070	$88,410	$89,460	$89,460
		+47%	+50%	+50%	+50%	+51%	+51%	+51%
		Benefits	Benefits	Benefits	Benefits	Benefits	Benefits	Benefits
	Length of visit (h): Hourly consultant fee	Best Available Estimate						
		$250	$250	$250	$250	$250	$250	$250
*XSEDE support services*								
Training services	Hourly online	Conservative Estimate: $9.98/h; Best Available Estimate: $95/h						
	Hourly live	Conservative Estimate: $11.29/h; Best Available Estimate: $95/h						
	Badge value	Conservative Estimate and Best Available Estimate: $1000						
Helpdesk services	Average ticket rate	Conservative Estimate and Best Available Estimate						
		$22.00/ticket (PY4–PY5)		$15.56/ticket (PY6–PY8)			$22.00/ticket (PY9–PY10)	
Extended consulting	Consulting hours at: National average annual salary for a computational scientist	Conservative Estimate:						
		$115,580	$116,320	$119,570	$123,850	$127,460	$130,890	$130,890
		+ 47%	+ 50%	+ 50%	+ 50%	+ 51%	+ 51%	+ 51%
		benefits	benefits	benefits	benefits	benefits	benefits	benefits
	Consulting hours at: Hourly consultant fee	Best Available Estimate						
		$250	$250	$250	$250	$250	$250	$250
Annual XSEDE cost	Total annual XSEDE expenditures	$26,563,247	$21,036,395	$18,285,622	$19,561,588	$19,993,698	$20,188,776	$19,571,663

#### XSEDE impact on research—non-financial data

We include one set of data other than financial data that sheds light on XSEDE’s role in affecting the mechanisms of science. This is data about the number of accounts of people using XSEDE generally and the number of accounts of people using a new cloud system that was implemented in 2016, called Jetstream. These data were collected by XSEDE accounting systems and are not published elsewhere.

## Results

### ROI_proxy_ estimates

**Fig. 5:**
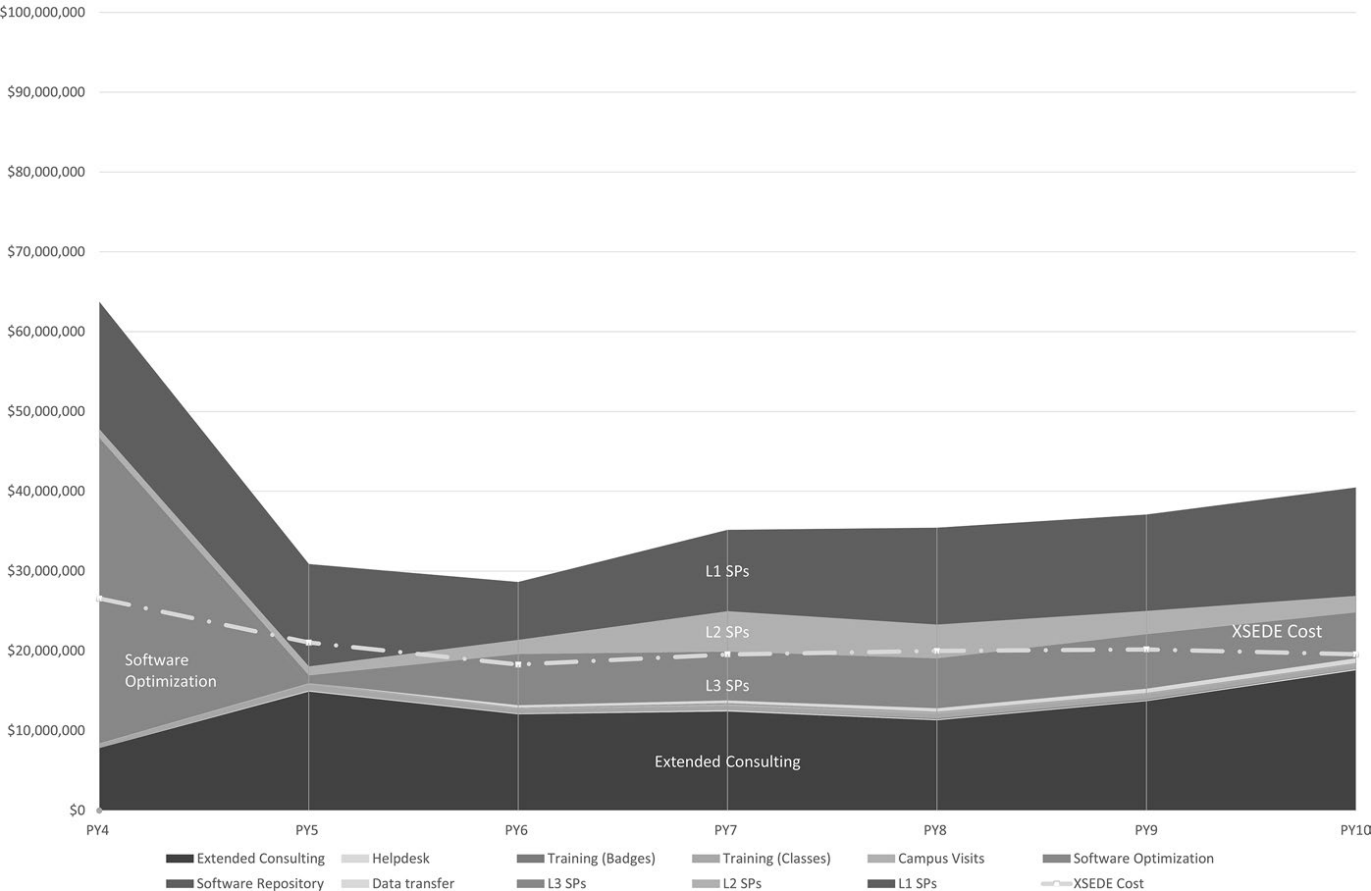
Values of services and costs of XSEDE for Conservative Estimate of ROI_proxy_

**Fig. 6:**
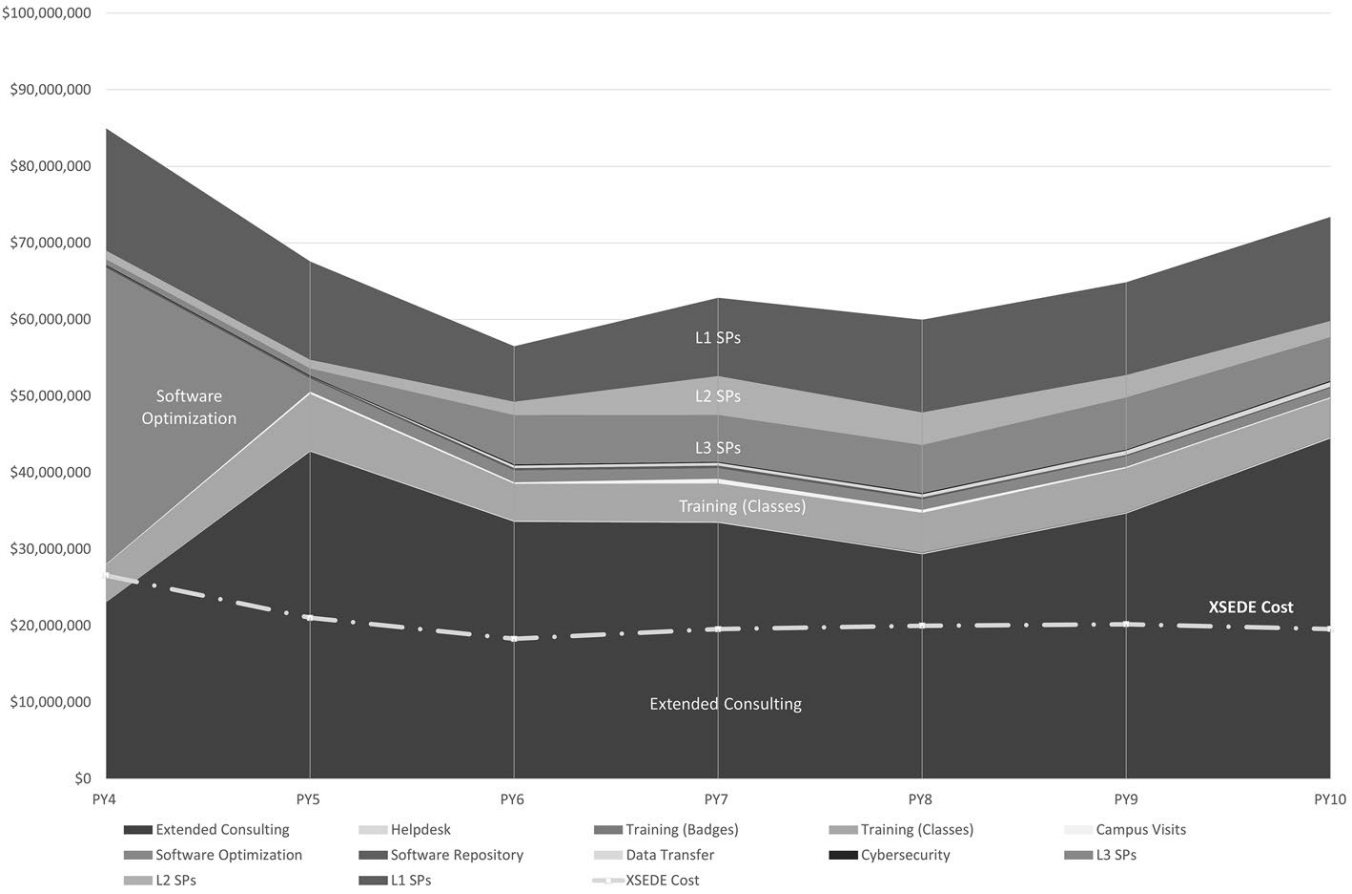
Values of services and costs of XSEDE for Best Available Estimate of ROI_proxy_

Table 3 and Figs. 5 and 6 present the Conservative Estimate and the Best Available Estimate of ROI_proxy_, with the valuations and costs underlying these estimates. More detailed views of data (means and assessment of variability of data) are provided in “Appendix 1.” Based on comparisons with other published papers about ROI for cyberinfrastructure, we believe that we have assembled the largest dataset in existence for return on investment in cyberinfrastructure (Costa et al., 2022; Wernert et al., 2022).

### XSEDE impact on research—non-financial data

*Adoption of cloud computing and expansion of XSEDE user community*. Table 4 presents data from XSEDE’s accounting system showing growth in user accounts on XSEDE as a whole and growth in the use of Jetstream, the first cloud system funded by the NSF for general purpose research.

**Table 3:** XSEDE service valuation and ROI_proxy_ estimates

Value/cost category	PY4	PY5	PY6	PY7	PY8	PY9	PY10	Mean
	Jul14–Jun15	Jul15–Aug16	Sep16–Aug17	Sep17–Aug18	Sep18–Aug19	Sep19–Aug20	Sep20–Aug21	PY4–PY10
*Conservative Estimate valuations*								
Operational and administrative support for SPs	$17,808,423	$14,876,262	$15,420,031	$21,400,550	$22,628,757	$21,822,633	$21,390,922	$19,335,368
Scientific software and cyberinfrastructure system administration	$37,569,060	$149,879	$490,383	$644,185	$649,596	$718,784	$754,076	$5,853,709
XSEDE support services	$8,369,297	$15,841,981	$12,705,431	$13,135,086	$12,143,088	$14,557,346	$18,318,678	$13,581,558
XSEDE total valuation using Conservative Estimates	$**63,746,780**	$**30,868,122**	$**28,615,845**	$**35,179,821**	$**35,421,441**	$**37,098,763**	$**40,463,676**	$**38,770,635**
*Best Available Estimate valuations*								
Operational and administrative support for SPs	$17,951,280	$15,019,119	$15,562,888	$21,543,407	$22,771,614	$21,965,490	$21,533,780	$19,478,225
Scientific software and cyberinfrastructure system administration	$39,060,526	$2,256,585	$2,397,170	$2,667,780	$2,420,530	$2,295,630	$2,120,664	$7,602,698
XSEDE support services	$27,945,823	$50,279,099	$38,536,934	$38,610,200	$34,763,627	$40,579,183	$49,710,645	$40,060,787
XSEDE total valuation using Best Available Estimates	$**84,957,629**	$**67,554,803**	$**48,103,052**	$**62,124,720**	$**61,259,938**	$**64,840,303**	$**73,365,089**	$**66,029,362**
*Annual costs*								
XSEDE total annual expenditures	$**26,563,247**	$**21,036,395**	$**18,285,622**	$**19,561,588**	$**19,993,698**	$**20,188,776**	$**19,571,663**	$**20,742,998**
Conservative Estimate of ROI_proxy_	**2.40**	**1.47**	**1.56**	**1.80**	**1.77**	**1.84**	**2.07**	**1.87**
Best Available Estimate of ROI_proxy_	**3.20**	**3.21**	**3.09**	**3.21**	**3.00**	**3.21**	**3.75**	**3.24**

Conservative Estimate and Best Available Estimate of XSEDE service value, XSEDE annual costs, and ROI_proxy_ estimates

Boldfaced values represent subtotals, totals, or overall values

**Table 4:** Growth in total number of accounts on XSEDE-supported L1 SPs and number of accounts on the Jetstream cloud system

Account type	Last PY before Jetstream 7/1/2015	Jetstream available 9/1/2016	End of PY10 8/31/2021
Accounts on all XSEDE resources	7414	8916	11,047
Jetstream cloud accounts		588	2559

## Discussion

This discussion does not consider the value of the actual outcomes and outputs of XSEDE, which are considerable and documented elsewhere (e.g. Towns et al., 2014; XSEDE, 2022a). Research enabled by XSEDE working together with SPs ranges from research leading to regulatory changes protecting stock markets against crashes (O’Hara et al., 2014) to research recognized by the awarding of two Nobel Prizes (Mahendran et al., 2022). XSEDE has supported other research with total funding of more than $3.6B during PY6-PY10 (XSEDE, 2022a). During that time period XSEDE was used in research resulting in 590 pieces of computer software, 23 patents, and 5 licenses of new technology (extrapolating from data in Chityala et al. (2022a, 2022b). Research supported by XSEDE through its initial 10 years has led to the creation of more than 20,000 peer-reviewed published papers (based on summation of data in XSEDE reports XSEDE 2022). With the basic value of XSEDE’s function hopefully made clear, the remainder of this discussion includes three sections: ROI_proxy_ values; the cost-effectiveness of XSEDE; and XSEDE’s impact on mechanisms of scientific research.

### Discussion of ROI_proxy_ values

“Is the US government getting a good deal on its investment in XSEDE?” is a simple and important science policy question. With the data presented here, we can offer a clear “yes.” The two mean estimates taken together—the Conservative Estimate of 1.84 and Best Available Estimate of 3.24—suggest that XSEDE is delivering services in ways that are highly financially effective.

It is in discussions of matters such as government funding policies for research that the Conservative Estimates of ROI_proxy_ are most useful. These estimates are *so* conservative that it is difficult to argue with them as a lower bound on value of investment in XSEDE. That provides a strong basis for discussion of funding policy that is well informed by data. [Conservative estimates derived from earlier and smaller datasets were discussed from the perspective of cyberinfrastructure professionals in papers from 2015 (Stewart et al., 2015) to 2022 (Stewart, Costa, et al., 2022).]

ROI analyses have already influenced NSF policy and funding strategies. Prior to the 2015 Stewart et al. paper on ROI for XSEDE (Stewart et al., 2015) there was considerable discussion within the US research community and the NSF about the structure of and funding for XSEDE. In particular, the NSF was seriously considering breaking XSEDE up and returning to the supercomputer center model of the 1980s. In 2015 Stewart et al. (2015) we showed that XSEDE was financially more efficient for the NSF than a return to the 1980s supercomputer center model would be. This and other factors put an end to discussion of reverting back to an NSF centers model. (This information is not published; the authors include leaders in the XSEDE project and we were privy to these discussions.) Indeed, in 2016 the NSF extended funding for the XSEDE project from the initial award of 5 years to a planned 10 years total. NSF policy requires a re-bidding of grants for facilities such as XSEDE at least once every 10 years. (XSEDE was extended to an 11th year as an exception and as a result of a number of unusual logistical factors.) The structure of the services that will come after XSEDE is very much like the structure of XSEDE (National Science Foundation, 2021). ROI analyses of XSEDE have helped inform these major policy decisions.

Overall ROI_proxy_ has remained relatively stable, with a gradual increase over time since PY7 (2017). One factor in this is that awareness of the impending end of XSEDE operations, originally scheduled for 31 August 2021, drove to completion some extended consulting projects that had been going on for a long time, an effect that one can see in a jump in valuation of XSEDE services for extended consulting. This contributes to an overall increase in ROI_proxy_ from PY9 to PY10. There is a clear and strong increase over time in the value of XSEDE to L3 SPs, part of which is related to the increase in the number of L3 SPs, as shown in Fig. 2. This jump in the number of L3 SPs was the result of a concerted effort by XSEDE staff, and the continued involvement of these organizations as L3 SPs throughout the project indicates the value of this status. Two factors underlie the decrease in total measured value of XSEDE services (and thus overall ROI_proxy_ values). One is the exceptional event of XSEDE’s work with LIGO, which contributed $37,500,000 to the valuation of XSEDE software optimization efforts. This value is included in the ROI_proxy_ values for PY4. This is the year that software improvements were completed and the year in which it would have been necessary to purchase additional systems hardware in the absence of these improvements. This activity was indeed exceptional. The LIGO project, with the assistance of XSEDE, verified Einstein’s previously untested theory of gravitational waves. This event happening in PY4 leads to a step function decrease in the annual valuation of XSEDE services from PY4 to PY5. There are decreases in the value of XSEDE from PY4 through PY6, particularly a decrease in the value of XSEDE to L1 and L2 SPs. During this time the original NSF grant award for XSEDE, which spanned PY1 through PY5, was transitioning to the NSF grant award formally called XSEDE2. There was a lower average annual budget for XSEDE2 than XSEDE, and a good deal of staff turnover associated with the transition. There is therefore good reason to believe that there was a real decrease in the value of XSEDE as perceived by SPs from PY4 through PY6.

There are interesting and important commonalities across years in the factors contributing to the total value assessed for XSEDE. Most particularly important in contributing to the total value estimated for XSEDE were the services offered to SPs and the XSEDE extended consulting services offered to PIs and their research groups. More realistic costs for the value of expert system administration and consulting services contributed to the considerably higher values overall for Best Available Estimates of ROI_proxy_, as did the inclusion of a more realistic representation of the value of systems hardware not needed because of XSEDE efforts to speed up software.

It would be helpful in interpreting the figures presented here if we were able to calculate something like a 95% confidence interval around estimates of ROI_proxy_. As discussed in “Appendix 1,” this is simply not possible, although measures of variability of samples are included in that appendix. One potentially useful metric in interpreting our analyses is the sensitivity of estimates of ROI_proxy_ to changes in the total assessed value of XSEDE services. If one considers a $1,000,000 variation in the average annual estimated value of XSEDE services, the result is a change of 2.6% in the Conservative Estimate of ROI_proxy_ and of 1.3% in the Best Available Estimate. $1M is equivalent to a change in estimated effort of about 5 FTE-years for the Conservative Estimate valuations and 4 FTE-years for the Best Available Estimate. Much of the most important contributors to ROI_proxy_ values overall depend on estimates made by individuals. We have confidence in the reasonableness of such responses, based on the expertise of the respondents, because PIs receiving XSEDE allocations are generally also individuals who have received federal research funding. Writing successful proposals requires the ability to accurately estimate the effort required to complete research tasks. The people who provided these effort estimates can thus be expected to be well-practiced and precise in such estimations. Indeed, individuals who responded to our surveys on behalf of SPs seemed to indicate more confidence in their ability to estimate effort than we had even asked for. In our survey instrument we requested estimates of effort to the nearest 0.25 FTE years. We routinely received FTE-year estimates to a precision of 0.01 or even, in some cases, 0.001.

As a methodological note, there was no “cooking the books” in our analyses. We had discussions and reached consensus about appropriate valuations for both estimates for ROI_proxy_, and only after reaching consensus on valuations did we make our first overall calculations. For the Best Available Estimate calculations in particular, we made some judgment calls that could seem arbitrary, such as the use of $1,000,000 for the value of a successful cyberattack, well under published figures. Even in the Best Available Estimate, we approached valuation with a certain conservatism. What one can see from the overall calculations is that individual valuations such as this do not have a strong effect on the overall outcome of the analysis. In addition, once the valuations were set, we never went back and adjusted valuations. The results were whatever they turned out to be.

Notwithstanding the limitations of statistical approaches to reliability, we are confident that whatever the ROI for the US government is in terms of investment in XSEDE services, it is greater than 1.0. Our estimates of ROI_proxy_ are likely reasonable to one significant digit, and maybe two, but certainly not beyond that. Perhaps the most valuable indicator of the reliability and robustness of our estimates of ROI_proxy_ is to be found in the range of estimated values, in other words between 1.87 and 3.24. These two estimates are based on very different sets of value equivalents for the services offered by XSEDE. It would be too much to say that this range constitutes any sort of statistical bound on the most reasonable estimate of the government’s Return on Investment (ROI) in XSEDE services, but whatever the best real value of these services is, it should be near to this range.

### Why is XSEDE cost-effective?

*XSEDE was designed from its inception to be a unified, learning, and evolving organization.* From the planning stages on, XSEDE developed a process for documenting processes and then modifying those processes over time (Towns et al., 2014). Documented plans and tips on how to resolve various kinds of unusual situations based on the accrued experience of hundreds of people help XSEDE operations, including change management, run effectively and efficiently. As a result, there is very little effort spent recovering from operational errors.

*XSEDE’s virtual structure facilitates cost-effectiveness.* XSEDE was designed to be a national virtual organization bringing together the best experts available but engaging most of them only part-time: XSEDE currently employs more than 200 people but a minority of its staff members work full-time for XSEDE. Persons whose skills are important to the XSEDE mission are generally employed by XSEDE for the amount of effort needed by XSEDE. This is particularly important in XSEDE extended consulting, where many experts in a large variety of science disciplines work part-time for the project.

*XSEDE achieves significant leverage of staff time not paid for by XSEDE.* XSEDE has created a community of practice and leveraged efforts of staff at universities and colleges whose salaries are not paid from the XSEDE budget. For example, XSEDE has fostered the creation of a large community of “XSEDE Campus Champions”—more than 700 individuals, on the campuses of more than 300 colleges or universities, who provide information dissemination and support services for XSEDE (2021)—without financial outlay from XSEDE aside from very small incentives such as funding for conference attendances for a few Campus Champions. In addition, L2 and L3 SPs are affiliated with XSEDE for a number of reasons, including a desire to publicize their services, a desire to stay in touch with the US cyberinfrastructure community, and/or a desire to use XSEDE services (XSEDE, 2020). The organizations that employ and fund people who work as Campus Champions and as staff in L2 and L3 XSEDE SPs are not doing this out of charity. Such investments in staff time are perceived to help organizations make more effective use of XSEDE services. Campus Champions perceive this status to aid professional development (Brazil et al., 2019). At the same time, such staff also contribute services, in support of XSEDE activities, that are not funded by XSEDE.

XSEDE staff sometimes subsidize XSEDE by putting forth effort on their own time—beyond their funding levels and beyond the NSF definition of a standard workweek. This is the personal experience of authors of this paper and a comment we have heard often from colleagues. This extra effort is often a result of interest in and dedication to the value of the scientific work that XSEDE supports. To the extent that individuals or other entities contribute effort to services that aid the operation of XSEDE without those services being funded by the NSF, that improves the US government’s ROI for XSEDE.

### XSEDE’s impact on mechanisms of scientific research

As intended by the NSF when it put out the solicitation that led to XSEDE’s creation, XSEDE has accelerated research by making services available that are otherwise not generally available to US researchers. This is in part the expected result of XSEDE having funding to provide very specialized services that would otherwise either not exist for US researchers or be extremely expensive from the standpoint of a university- or college-based researcher. But XSEDE has also effected changes in the mechanisms of scientific research and practices of the US research community through its strategies and services.

*Availability of excellent staff with diverse expertise.* As mentioned above relative to cost-effectiveness, XSEDE is able to contract, for modest portions of time, the skills of a large number of individual experts. Being an XSEDE staff member provides the opportunity to work with cutting-edge technology. The universities and colleges for which such individuals work view this as a positive, in addition to the funding they obtain through subcontracts to pay for a portion of the time of their staff. XSEDE is thus able to make available consulting expertise in a large variety of areas of specialty—more than any university or college could possibly afford when hiring individual full-time staff.

*Facilitating change in researcher practice.* XSEDE has played an important role in changing community practices in the use of advanced computing resources in research. Prior to XSEDE there was never a formalized process for bringing a new computational, storage, or visualization system online and making it available to the US research community. A particularly good example is seen in facilitating the adoption of cloud computing and increasing the total number of users of XSEDE. Prior to deployment of the Jetstream cloud system in 2016, XSEDE had never supported use of a cloud system. As of the end of PY10, 23% of the individuals who had accounts on an XSEDE-supported L1 SP had Jetstream accounts. Of individuals with Jetstream accounts, the vast majority had not used any XSEDE-supported resource prior to obtaining a Jetstream account (Hancock et al., 2019). Growth in total XSEDE users almost exactly parallels the addition of users with Jetstream accounts, and this growth was more rapid than it had been in the years directly prior to the deployment of Jetstream. The impact of XSEDE and Jetstream on each other was such that XSEDE fostered the adoption of Jetstream in terms of number of users and disciplinary diversity of users, and Jetstream drove an increase in the total number of users of XSEDE. These two factors together drove an increase in use of non-commercial cloud services by the US research community and increased the fraction of the US research community taking advantage of the NSF’s investment in XSEDE. [Jetstream is itself highly cost-effective (Stewart et al., 2019).]

*Facilitating research in an international emergency.* XSEDE played an important role in facilitating research related to the COVID-19 pandemic. In the spring of 2020, the US government fostered the creation of the COVID-19 HPC Consortium to support research on COVID-19. It includes 42 private- and public-sector partners. XSEDE put in place an allocation process for this consortium within days. This enabled the consortium to support more than 100 research projects that contributed to the US response to COVID-19 (Brase et al., 2022).

*Creating a reliable utility for research support*. XSEDE has become viewed as a “utility” aiding research. XSEDE is sufficiently reliable that researchers have made research plans and written grant proposals to government funding agencies based on the assumption that they would be able to depend on XSEDE and SP resources for advanced computationally based research. These research projects, with an aggregate budgetary value that dwarfs the total cost of cyberinfrastructure support ($38B as compared to $0.5B total for XSEDE and L1 SPs over 10 years), have been successfully completed while making use of XSEDE services. Furthermore, a majority of the PIs who received allocations for use of XSEDE and XSEDE-supported L1 SP resources indicated that they simply could not have done their research without these resources—indicating that XSEDE’s services are affecting research execution within the US and likely also the development of research questions and plans in the US (Hart, 2022). There are two events where it seems likely that the availability of XSEDE as a utility helped the government save significant amounts of money. While we do not know for certain, it seems likely that in the absence of XSEDE, the NSF might have fulfilled the LIGO request for an additional $75M in funding for its computational infrastructure. Certainly it would have been more expensive to set up an allocations operation for the COVID-19 HPC consortium had XSEDE’s system not been ready and available for use.

## Conclusion

We have presented data that show XSEDE services to be offered in financially effective ways relative to the US government’s investment in XSEDE. According to our best estimates, the US government has received an ROI of at least 1.87 for its investment in XSEDE services. This represents a conservative estimate and a lower bound on the actual ROI for such investments. An estimate based on what we consider to be the best available and most reasonable estimate of the value of XSEDE services results in an ROI of 3.24. These ROI estimates are above the 1.20 to 1.25 overall ROI value for public investment in research cited in a report from the United Kingdom (Frontier Economics Ltd, 2014). That is, the ROI for US government investment in XSEDE is better than the results of the most comparable analysis we have been able to find for public investment in innovation. This does not mean that XSEDE somehow and in general reduced costs; there was no prior and more expensive service. The data presented here strongly imply, however, that had the US government purchased a set of services equivalent to those delivered by XSEDE from the general US marketplace, it would have spent much more than it did to fund XSEDE.

It is not possible to establish an overall rate of return on investment in scientific infrastructure within just a few years of such an investment. Public returns on knowledge are sometimes direct and sometimes indirect, through long-term contributions to technology and quality of life. Indeed, as pointed out in Lane (2009), no single number can adequately capture the return on a public investment in research. Public returns tend to increase over time, as improvements that affect health and quality of life continue to accrue value steadily. The quantification of the value of XSEDE’s outputs and outcomes remains a task for the future. Approaches such as the Value Reporting Foundation’s reporting framework may be useful in such efforts (The Value Reporting Foundation, 2021).

While the financial concept of ROI has been used in earlier analyses of cyberinfrastructure facilities in general and XSEDE in particular, these reports were focused on this matter from the perspectives of practitioners and federal funding agency personnel. The analysis presented here focuses on ROI analysis as a tool for understanding mechanisms of science and constitutes a potential model for future use in scientometric studies of mechanisms of science. While we focus here on a cyberinfrastructure project, the basic methodology we have used can be extended to other types of facilities supporting research.

We certainly have not addressed the large-scale questions about relationships between investment and innovation posed by Schumpeter 110 years ago (Schumpeter, 2003). However, we have shown that it is possible to assess the value delivered as a result of federal investment in cyberinfrastructure on a year-in, year-out basis in a reasonable and quantitative way that creates information useful to scientists, policy-setters, politicians, and the voting public. We believe we have also demonstrated the utility of concepts drawn from accounting as tools with which to analyze the mechanisms of science and innovation and thus to enable a better quantitative understanding of the mechanisms of scientific research. We hope that this paper, in addition to shedding some light on the value of one US government investment in research infrastructure, will spur additional interest in the use of accounting-based concepts in scientometrics research.

## Data Availability

Source data and a report describing analysis methodology are available online (Costa et al., 2022; Wernert et al., 2022).

## Appendix 1: ROI_proxy_ estimation details

Tables 5 and 6 provide details for the calculation of the Conservative Estimate and the Best Available Estimate for ROI_proxy_. There is no good way to calculate confidence intervals around estimates of ROI_proxy_. These estimates are based on three qualitatively different kinds of numbers: market valuations for services that are not a sample from a population of values; counts of service units that are known essentially exactly; and human estimates of the value of services supplied by XSEDE. Particular issues that limit the meaningfulness of confidence intervals around the human estimates of value are:

- *L1 SPs*. We have an essentially exhaustive survey of responses for the entire population of L1 SPs; we are not sampling data and there is no extrapolation back to some unknown population mean value. Also, the relationships between SPs and XSEDE are highly variable. Some are host to one resource supported by XSEDE, others host multiple resources.
- *L2 SPs*. The relationships between the L2 SPs and XSEDE are extremely variable; they range from consulting groups to system providers, with very little consistency in what XSEDE services they use and value.
- *L3 SPs*. For the L3 SPs we sample well over half of the SPs but not all, so it is sensible to estimate the variability of the mean value for the L3 SPs.
- *PI assessments of the value of extended consulting services*. The distribution of values is not normal because we set an upper limit of 24 person-months for the value of XSEDE to a particular PI.

Table 7 provides statistics on number of responses, population size, and standard deviation within the dataset that will allow the reader to get a sense of the variability in the data derived from human estimates.

**Table 5:** Details for Conservative Estimate of ROI_proxy_

Category of value	PY4	PY5	PY6	PY7	PY8	PY9	PY10	Mean
	Jul14–Jun15	Jul15–Aug16	Sep16–Aug17	Sep17–Aug18	Sep18–Aug19	Sep19–Aug20	Sep20–Aug21	PY4–PY10
*Operational and administrative support for SPs*								
Service to L1 SPs	$15,948,215	$12,818,846	$7,241,488	$10,207,774	$12,118,883	$12,084,090	$13,571,076	$11,998,625
Service to L2 SPs	$1,104,111	$1,107,633	$1,753,718	$5,056,986	$4,221,851	$2,926,801	$2,081,598	$2,607,528
Service to L3 SPs	$756,097	$949,783	$6,424,825	$6,135,790	$6,288,023	$6,811,742	$5,738,248	$4,729,215
Cybersecurity	$0	$0	$0	$0	$0	$0	$0	$0
Subtotal	$**17,808,423**	$**14,876,262**	$**15,420,031**	$**21,400,550**	$**22,628,757**	$**21,822,633**	$**21,390,922**	$**19,335,368**
*Services related to scientific software and cyberinfrastructure system administration*								
Data transfer	NA	NA	$327,700	$359,300	$422,300	$557,400	$615,700	$456,480
Software repository	$29,393	$33,969	$41,961	$48,259	$35,945	$26,348	$23,376	$34,179
Software optimization	$37,500,000	$0	$0	$0	$0	$0	$0	$5,357,143
Campus visits	$39,667	$115,910	$120,722	$236,626	$191,351	$135,036	$115,000	$136,330
Subtotal	$**37,569,060**	$**149,879**	$**490,383**	$**644,185**	$**649,596**	$**718,784**	$**754,076**	$**5,853,709**
*XSEDE support services*								
Training services	$546,680	$828,359	$549,190	$585,134	$727,745	$740,416	$598,950	$653,782
Helpdesk services	NA	$101,156	$112,281	$125,320	$120,077	$120,208	$145,024	$120,678
Extended consulting	$7,822,617	$14,912,466	$12,043,960	$12,424,632	$11,295,266	$13,696,722	$17,574,704	$12,824,338
Subtotal	$**8,369,297**	$**15,841,981**	$**12,705,431**	$**13,135,086**	$**12,143,088**	$**14,557,346**	$**18,318,678**	$**13,581,558**
*Totals*								
XSEDE valuation	$**63,746,780**	$**30,868,122**	$**28,615,845**	$**35,179,281**	$**35,421,441**	$**37,098,763**	$**40,463,676**	$**38,770,635**
XSEDE cost	$**26,563,247**	$**21,036,395**	$**18,285,622**	$**19,561,588**	$**19,993,698**	$**20,188,776**	$**19,571,663**	$**20,742,998**
ROI_proxy_	**2.40**	**1.47**	**1.56**	**1.80**	**1.77**	**1.84**	**2.07**	**1.87**

Boldfaced values represent subtotals, totals, or overall values

**Table 6:** Details for best available estimate of ROI_proxy_

Category of value	PY4	PY5	PY6	PY7	PY8	PY9	PY10	Mean
	Jul14–Jun15	Jul15–Aug16	Sep16–Aug17	Sep17–Aug18	Sep18–Aug19	Sep19–Aug20	Sep20–Aug21	PY4–PY10
*Operational and administrative support for SPs*								
Service to L1 SPs	$15,948,215	$12,818,846	$7,241,488	$10,207,774	$12,118,883	$12,084,090	$13,571,076	$11,998,625
Service to L2 SPs	$1,104,111	$1,107,633	$1,753,718	$5,056,986	$4,221,851	$2,926,801	$2,081,598	$2,607,528
Service to L3 SPs	$756,097	$949,783	$6,424,825	$6,135,790	$6,288,023	$6,811,742	$5,738,248	$4,729,215
Cybersecurity	$142,857	$142,857	$142,857	$142,857	$142,857	$142,857	$142,858	$142,857
Subtotal	$**17,951,280**	$**15,019,119**	$**15,562,888**	$**21,543,407**	$**22,771,614**	$**21,965,490**	$**21,533,780**	$**19,478,225**
*Services related to scientific software and cyberinfrastructure system administration*								
Data transfer	NA	NA	$327,700	$359,300	$422,300	$557,400	$615,700	$456,480
Software repository	$243,250	$268,000	$324,000	$369,500	$269,250	$197,250	$175,000	$263,750
Software optimization	$38,747,276	$1,678,585	$1,565,470	$1,350,980	$1,350,980	$1,350,980	$1,214,964	$6,751,319
Campus visits	$70,000	$310,000	$180,000	$588,000	$378,000	$190,000	$115,000	$261,571
Subtotal	$**39,060,526**	$**2,256,585**	$**2,397,170**	$**2,667,780**	$**2,420,530**	$**2,295,630**	$**2,120,664**	$**7,602,698**
*XSEDE support services*								
Training	$4,924,990	$7,443,915	$4,848,895	$5,044,880	$5,299,800	$5,808,975	$5,105,095	$5,496,650
Helpdesk services	NA	$101,156	$112,281	$125,320	$120,077	$120,208	$145,024	$120,678
Extended consulting	$23,020,833	$42,734,028	$33,575,758	$33,440,000	$29,343,750	$34,650,000	$44,460,526	$34,460,699
Subtotal	$**27,945,823**	$**50,279,099**	$**38,536,934**	$**38,610,200**	$**34,763,627**	$**40,579,183**	$**49,710,645**	$**40,060,787**
*Totals*								
XSEDE valuation	$**84,957,629**	$**67,554,803**	$**56,496,992**	$**62,821,387**	$**59,955,771**	$**64,840,303**	$**73,365,089**	$**67,141,711**
XSEDE cost	$**26,563,247**	$**21,036,395**	$**18,285,622**	$**19,561,588**	$**19,993,698**	$**20,188,776**	$**19,571,663**	$**20,742,998**
ROI_proxy_	**3.20**	**3.21**	**3.09**	**3.21**	**3.00**	**3.21**	**3.75**	**3.24**

All values here are different than the Conservative Estimate values except service to SPs, data transfer, helpdesk services, and cost of XSEDE

Bold-faced values within tables represent subtotals or totals

**Table 7:** Mean, standard deviation, responses, and population for valuations of XSEDE provided by representatives of L1, L2, and L3 SPs and by PIs who received XSEDE extended consulting services

Measure	PY4	PY5	PY6	PY7	PY8	PY9	PY10
*Level 1 SPs*							
Mean	$245,880	$193,459	$141,846	$198,383	$244,874	$235,980	$252,636
SD	$317,631	$238,902	$216,966	$296,425	$333,547	$283,435	$314,998
Responses/population	3/5	4/4	4/4	4/4	4/4	4/4	4/4
*Level 2 SPs*							
Mean	$16,783	$17,052	$13,953	$46,173	$38,420	$33,702	$21,627
SD	$19,501	$19,813	$14,554	$26,834	$44,319	$35,292	$28,504
Responses/population	4/5	5/5	2/8	8/9	6/8	7/7	5/7
*Level 3 SPs*							
Mean	$7,992	$9,860	$24,408	$21,607	$19,838	$17,976	$17,027
SD	$14,226	$11,528	$24,669	$19,585	$24,337	$13,226	$13,229
Responses/population	3/5	7/7	13/21	13/23	17/25	16/26	17/26
*Extended consulting*							
Mean	$120,348	$252,754	$188,187	$258,847	$251,006	$247,055	$279,172
SD	$97,254	$119,743	$141,589	$119,711	$144,889	$151,279	$136,392
Responses/population	12/65	24/59	33/64	25/48	20/45	23/54	22/62
